# James Taylor, M. D., on Filling Teeth

**Published:** 1851-04

**Authors:** James Taylor


### SELECTED ARTICLES.

### ARTICLE VII.

### JAMES TAYLOR, M. D., *on Filling Teeth.*

[Continued from January No.]

BEFORE I give any special directions as it regards the finishing of this important operation, I will take up the other classification of cavities which I have given. I do this because the finishing of a plug requires about the same manipulation in all cases. The next locality of cavity is the second in my classification, or the labial. This location of disease is not of so frequent occurrence as the central or approximal; yet of so frequent occurrence as to demand a separate description. They appear to be the result of two or three physical conditions of the teeth and fluids of the mouth. First, we have of the teeth, and especially of the inferior molars, and sometimes, it is true, of the superior—an extension, as it were, of the transverse suture running from the center of the tooth to the labial surface; so that the tooth appears to be divided by a medial line. Decay may take place at any point on this line, and be, in its general character, the same as that originating from the same cause, and located on the grinding surface of the tooth. But as we advance forward, and find cavities on the labial surface of the teeth, we generally find them located nearer the margin of the gum, and often dipping beneath its free edge. The thin edge of enamel which terminates at the neck of the tooth has been destroyed by some corrosive fluid, or the gum has receded, leaving the bony structure exposed, so that we have a softening of the dentine, and a shape of cavity running transversely of the tooth, and if the disease has been permitted to extend itself, we shall have a cavity the shape of a half circle, with its base formed by the enamel and bone, and the circular line extending around under the gum. This decay is not, however, always thus circumscribed, for at times it may be found entirely confined to the enamel portion of the tooth, forming a round pin-head cavity. The teeth most subject to this form of decay are, I think, the upper front incisors, next in order the lateral, then the canine and first bicuspids inferior, then the canine superior, and molars inferior, &c. &c. The preparation of all these cavities cannot well be accomplished with the same instruments or in the same manner. The small round pin-head cavities, after having been thoroughly examined, and the enamel removed which covers the decay, can be very neatly and expeditiously prepared for filling, with a small burr-head drill. If the cavity does not penetrate deep into the tooth, the burr-head should be flattened to represent a half sphere. (They are in too common use to need a description.) A drill of this description will, if selected of the right size, make just such a shaped cavity as is most desirable when the decay is not partly covered by the gum. These drills should be sharp, for a drill which cuts rapidly hurts no more, if not less, than one which cuts more slowly, or requires considerable pressure to make it cut at all. The only objection to the use of this instrument is, that it produces, generally, more pain than the cutting instruments. When this is the case, use the excavators to remove the carious portion; and then shape the cavity with the drill.

When the cavity runs beneath the gum, the drill is not so admissible, first, because the cavity is not so round, and second, as the gum may be in the way, or too much of the tooth may have to be cut away to make the cavity round. The shape of cavity already described, with its base extending across the tooth, and its circle extending under the gum, is most desirable; because the decay takes somewhat this form. We will find, generally, no difficulty here in making the wall of the cavity, which forms the base, of sufficient depth to well secure a filling; but that portion which extends beneath the gum will require more care, for as the tooth diminishes in size as we advance on its neck, so will the cavity diminish in depth. If the decay should be deep, the nerve lies close at hand; if not, the upper border of cavity must be shallow, and this is the first part to be filled. In the preparation, then, of these cavities, we would, after removing that portion of decay most exposed to view, carefully, with a suitably shaped excavator, cut away the decay around the border of the cavity underneath the margin of the gum, and form this portion, slightly beveling inward, so as to enlarge at this part a very little the cavity within. This is done so that the first fold or block of gold, when forced well into its place, may be securely retained. A grooved necked cavity is not that which is desired, but simply a beveled condition of that portion of the wall which is overlapped by the gum. If this is formed with an instrument, the cutting edge of which comes to right angles, a grooved surface to the wall of the cavity will be easily avoided. No condition of decay requires more careful manipulation than the one now under consideration. The plug is more exposed to view; also to the action of the brush, the daily use of which may be requisite to prevent the further progress of the disease. We are often, too, exceedingly annoyed with extreme sensibility of tooth bone.

These cavities, when located on the incisors and bicuspids, above or below, although presenting difficulties in the proper formation of the cavity, yet in the filling itself, these difficulties are much increased as we advance back in the mouth; so much so, indeed, that the dentes sapientiæ, superior or inferior, when presenting labial cavities, may be considered among the most difficult to fill of any in the mouth. These teeth, however, when much decayed, and the others in anything like good condition, are scarcely worth an effort to preserve, yet their preservation must depend on their utility for mastication and other purposes, which must be decided by the judgment of the operator.

The extent of disease in these teeth, (the molars and dentes sapientiæ,) will control, as a matter of course, the form of cavity; sometimes a round, and at other times an oblong, shape will be presented; frequently the decay extending from approximal to approximal surface; and when this is the case, great care is necessary in the formation of the cavity. That portion of the cavity having its walls formed by the neck of the tooth and the solid enamel and bone which forms the cutting surface, will present no difficuty in a proper adaptation to retain a plug; but the posterior wall, also anterior, will require a little more care, especially when the cavity runs nearly across the tooth, and for this reason: if these are cut with the plane of the wall pointing toward the center of the tooth, we shall have a cavity running transversely with the tooth, far larger on its outer circle than within, so that the plug would have to be retained entirely by the side walls of the cavity, and the tooth left subject to that very condition of things which we so often see occur after a filling of this kind, viz. a continued progress of decay running around these teeth, as it were. The cavity not having been properly formed, the gold is not so compacted as to exclude the moisture; hence the bone continues to soften at these points. The posterior wall should, therefore, be made so as to apparently lean forward, and the anterior running in, so as to maintain the size of the cavity within. If these suggestions are properly observed, the introduction of the gold will be rendered less difficult, and the filling more secure.*

Having alluded to the shape of cavities most frequently occurring in the localities under consideration, we will next speak of the introduction of the foil; and first in those last described. Here we take, as an illustration, an inferior posterior molar, having a cavity extending quite from approximal to approximal

* I have occasionally filled these teeth, (the inferior molars,) when the decay extended on to both approximal surfaces. I have then shaped the walls of the cavity at the neck of the tooth, and that formed by the solid portion of the crown above, beveling inward, enlarging the cavity a little within its orifice. This shape of cavity is particularly obtained at the posterior extreme of decay. Where the foil is first wedged in, and, to a great extent, consolidated, before the anterior portion of the cavity is filled, the first gold here introduced, forms the posterior wall for the last part of the plug, and the enlarged condition of the cavity within, prevents the first portion of the filling from being displaced by the pressure made in introducing the balance of. the plug. The condition of the cavity is such, extending almost half round the tooth, that the plugging instruments can be introduced on a line with the length of the cavity, and the gold thus compacted into this enlarged cavity without any difficulty.

surface. The cavity having been shaped as described, the first difficulty which presents itself, is easy access to the cavity with suitable instruments for filling. The second, exclusion of moisture. The first difficulty can generally be overcome by the use of the speculum; and when this is used, the second difficulty is also overcome at the same time. To secure both objects at the same time with this instrument, we would place on the tongue the end of a napkin folded lengthways, and so as to give one corner as the folded end; this I would throw around the dentes sapientiæ, placing the end next the cheek. The speculum is then secured on this, so that the tongue is held in place, and the folded end of the napkin pressed against the cheek. The bars forming the speculum should be somewhat wider apart than those used when filling central cavities; flattened and widened out, and slightly turned up, so as to form a cheek-holder as well as tongue-holder; or the cheek can be held away with the ordinary pearl cheek-holder, yet this will not so effectually secure a napkin to absorb the saliva.

All admit the importance of keeping the cavity dry, and all must admit the difficulty sometimes experienced in accomplishing this object. That method which practice has made most available, should, of course, be adopted by every operator. I therefore merely give that method of which I most usually avail myself.

These preliminaries having been gone through with, and the cavity dried, the next thing is the insertion of the gold. That method which accomplishes this object in the shortest time—filling up completely the cavity, will, I have no doubt, be considered the best.

This remark is applicable, especially when we remember that these cavities cannot be kept dry for an indefinite length of time. My former method, and one which I sometimes yet pursue, is done in the following manner: roll up, in a cylindrical form, as much or more gold than is necessary to fill the cavity; then fold up one end of this roll into a ball as large as can be forced into the cavity; then, with a sharp-pointed plugger, press this into its place, folding in as much of the roll thereafter as is possible. There are objections I know, to this method, for the quantity of gold first introduced may be too large to secure a perfect adaptation of the foil to every part of the cavity; or the mouth of the cavity may be choked up, as it were, before the interior is well filled. I have, therefore, of late, more frequently resorted to the plan already described, for the insertion of gold in the central cavities. I would have my blocks of gold so arranged that each would require slight compression with the plugging forceps to pass readily into the cavity. The plugging forceps here used are bent at near right angles. The first block used, however, here, would not always be the largest; but after placing the first block to its place, close the forceps, and using it then as an ordinary plugging instrument, force back the lower end of the block, (or that end within the cavity,) to the posterior wall, which, you recollect, is made slightly leaning forward; then, with one or two backward motions of the instrument, force the protruding end of the block out of the way of the next one to be introduced. Then, as each block is inserted, compress back against the first one introduced, completely filling up the cavity as you advance anteriorly, when, with a smaller pointed plugging instrument, a few folds of the strip to which the last block belongs, must be forced in. We are now ready for the compression, and if this can be used with considerable force, before the moisture has got about the gold, the success of the filling is certain.

The question here may be asked, is the compression which is necessary to be made, to compact these blocks, made with the plugging forceps? I answer, not always—yet, to a great extent, such is the case; but I always have ready one or two of such ordinary pluggers as are most apt to be needed, and occasionally use that which may be apparently demanded to arrange and force into place these blocks, also to make pressure around the border of the cavity, and all over the gold, to see if it is well compacted.

The plugging of these large labial cavities, when located far back in the mouth, with foil cut in strips and folded in with the plugging instrument, I have always considered a difficult, if not almost an impracticable operation. The time requisite to arrange these folds with an instrument, and at the same time compact them in the cavity, is generally longer than such cavities can be kept dry. After the blocks are prepared, they are much sooner inserted, and when in are far more systematically arranged, and to make the plug equally solid, do not require near the same amount of pressure.

The filling of these labial cavities seated above the gum and on the medial suture dividing the outer prominences of the tooth, require scarcely a separate description. When not large, they can generally be shaped with a drill, and filled with a few blocks, as described in such cavities when central. Those in the superior molars, requiring different means to hold off the cheek and keep the moisture from the cavity. To effect this, I use, when the posterior molar or dens sapientiæ is to be filled, the common pearl cheek-holder, holding the end of a folded napkin between this and the cheek, almost entirely filling and compacting my gold with the plugging forceps; but before displacing my cheek-holder, I always use considerable force with my compressing instruments. All these plugs which we have been considering, will first need, (after having been as far as possible consolidated,) the free use of the file, so that the gold may be leveled down and rounded to resemble the natural contour of the tooth, and so that the edges of the cavity may also be made smooth with the surface of the gold.

We come now to speak of the filling of those cavities on the labial surface of the incisors, canine and bicuspids—those located so far from the gum as to be entirely surrounded by healthy enamel, are generally round, and should be filled when the cavity is small, so as to be as little observed as possible. These are generally shaped with a drill, and when very small can be as well filled by taking four to six thicknesses of No. 4 or 6 gold foil, cut into a strip not wider than the breadth of cavity. The first fold of the gold should be carried to the bottom of the cavity, and each succeeding fold also carried as near the bottom of the cavity as possible, so that these several folds may, when compressed, be like so many wedges. After compression has been thoroughly made, the ends of these folds should be sufficiently prominent to require the use of the file to level the plug to the surface of the tooth.

Those cavities circling under the free edge of the gum, require a little different treatment. That portion of the cavity under the gum has not the same depth as that surrounded by the enamel, and is slightly beveled, as already described, so that the inner end of the first fold or the first block of gold, should be well wedged under the beveled wall.

This portion of the cavity should be first filled, because covered by the gum, and the pressure of the gold is necessary to keep the gum out of the way. If the cavity is large, I prefer the use of blocks for these fillings, and if small, I use the strips as already described, forcing the first fold under that portion of the wall of the cavity formed beneath the gum, and if the cavity is large, placing my first block in the same place, and finishing at that portion of the cavity where the border is surrounded by healthy enamel. We have here a cavity so formed that when the gold is well compacted, the filling cannot be dragged or worn out by the use of the brush, and the bone cannot well soften around the plug; at least this cannot well take place, unless the gum is absorbed so as to expose the bone above on the neck of the teeth. The shape of the outer surface of the plug should correspond with the shape of the tooth, and should be trimmed around the margin of the cavity under the gum, so that no projecting portions of the gold shall irritate the gum. If this should be the case, the same condition of the gum will soon be manifest as that caused by the presence of salivary calculi.

Small fissures are sometimes observed running across the superior incisors, apparently the result of an ill formation of the tooth itself, but which has been enlarged by a decomposition of the tooth substance. These should be excavated to suit the shape of the fissure, preserving the appearance of the tooth as well as possible, and then commencing at one end of the cavity, fill up so as to restore the shape of the tooth, and prevent further decay. The taste and judgment of the operator must decide the form of cavity most suitable to preserve the beauty of these important organs, so conspicuous in the mouth. Decay is sometimes found at the neck of the inferior incisors, canine and bicuspids; yet a further description of labial cavities is now unnecessary.

The next cavities in regular order, according to classification, are the approximal, yet, in their importance and frequency, they might well be placed first on the list. I presume that they embrace far more in number than all the rest, and that no location of cavity so much baffles a majority of operators. How frequently do we meet with young operators who succeed tolerably well in the central and some of the labial fillings, and yet scarce ever succeed in those now under consideration, and if so at all, only in those most favorably situated for filling.

There are quite a number of circumstances and physical peculiarities of the teeth to be taken into consideration, before we can treat of this operation as we ought. These cavities are located on the approximal surfaces of the teeth. The age of the patient, the nature of the decay, the extent of disease, the tooth or teeth to be filled, and the means to be used so that the cavity can be made accessible and the teeth filled. Shall they be separated by wedging or by filing? I think we shall find that these several circumstances will and should exert a due influence on the mode of operating. In our description, we take the teeth as we find them, decayed on their approximal surface, and we are to determine whether they can be saved by filling, and how this is to be best done. We find all the teeth subject to decay in this location, true, some but seldom, others very often; the superior incisors and bicuspids more frequently; the incisors and canine inferior less frequently; yet all are subject to this location of disease.

The age of the patient must have an influence in directing us in the first steps necessary to perform this operation aright.

The question might be asked, at what period of life are we most frequently called upon to fill the inferior incisors? The answer to this question would depend very much on the oversight of the parent, and whether children are brought in to have these teeth plugged when they should be. I am satisfied that in a majority of cases when these teeth decay on their approximal surface, that the disease commences before the twelfth year, or by that period of life we may expect the second molars to make their appearance. These remarks are made because it is thought that in early life they should be separated by wedging, and at this time they can be thus separated with less injury than in after life. What are the conditions which would demand the use of wedges instead of the file? If the decay has progressed so that the edges of the enamel have become brittle, the file is certainly indicated; and if, when this has been used, the space obtained is sufficient for the proper introduction of the gold, wedging is not necessary. If the teeth are much decayed and overlapping each other, and the use of the file would relieve the deformity, it is certainly indicated; but if, when this has been used so that the object is accomplished, and the enamel around the border of the decay is firm and solid, and the space is not sufficient, the use of the wedge is indicated.

If the decay is small, not deep-seated, and has not injured the appearance of the teeth, wedging is certainly admissible, and most particularly so, if the use of the file would cut away a portion of the border of the cavity necessary for the retention of the plug.

If the teeth are small at their necks, and only pressing against each other at their points, and these sound, with small decay, the wedge is indicated. I have thought that this particular condition of things was more favorable for the use of the wedge than any other. Such teeth, when filed, generally approximate and bring their filed surfaces together, because a shoulder is not admissible at their necks, so that they can be kept apart. Such teeth, when much filed, are often much disfigured, from the fact, that two approximating, some of the spaces are much widened. Take, for illustration, the central incisors pressing together at their points, the lateral incisors more disposed to rest against the canine teeth. The approximal surfaces are all decayed, and spaces are made with the file for filling. We will find the central incisors soon again pressing at their points; increasing the space between these and the lateral, about equal to the space made between the central, and if the lateral should still tend toward the canine, a still greater deformity will be created, the extent depending upon the space made between these and the canine. This condition of things, then, indicates very strongly the propriety of wedges. and I should not stop to know if my patient was fourteen or forty.

We will find such teeth very easily pressed apart—the alveolus not embracing so much of the root of these teeth as is the case in teeth of short crowns and thick necks.

What kind of wedges shall we use? This I think not really essential. The object is the separation of the teeth, and the requisite force can be applied by any of the three usual methods, and with care can be effected with safety by either. The gum elastic, it is true, unless judiciously applied, may exert the force too rapidly. The same thing, however, may be effected by the use of wood or cotton. The latter articles, however, I usually employ, because less unseemly between the teeth. If the carious portion is very sensitive, and I wish to destroy this sensibility, I use cotton, having upon it tannin and collodion, or creosote and extract of galls. The former I have of late found the most to be depended on. The collodion being soon evaporated, the cotton is left in a somewhat hardened ball, and not easily displaced.

My attention was first drawn to the use of the cotton for wedging the teeth apart, by a case I had in Port Gibson, Miss., some ten years since. Mrs. T., aged about thirty-five, had extensive decay on the posterior approximal surface of the second bicuspid, left side superior. The tooth had been filled, and the plug recently picked out. The tooth bone was exceedingly sensitive, and to allay this I applied daily, for a few days, a preparation of nut-galls with creosote on cotton. The space between the teeth was so small that I had to force the teeth slightly apart to get in my cotton with the preparation named.

I found the space widening daily, and by the time the tooth was ready to fill, the space was abundant to enable me to fill without the use of the file. When the object is a separation of the teeth, and no application to the decay is desired, I generally take soft wood, (pine,) and after introducing the first wedge, leave the balance to my patient, directing a renewal every morning and evening, until the requisite space is obtained; not letting the pressure be enough at any time to produce much soreness in the teeth. This will accomplish the object in from two to four or six days.

I avoid the use of arsenic in acute sensibility of tooth bone, unless I am absolutely driven to it, and this is very seldom; but above all other means, I far prefer the rapid use of a very sharp excavator. This is at once forced through the decay, when with two or three sudden revolutions around the cavity, the decay is mostly cut loose from the bone, and its sensibility in great measure paralysed. After this, time can be taken to complete the cavity as desired. There are but very few patients, of even twelve years of age, but what I ultimately get to submit to this operation. When such will not submit, and the other remedies fail, I would then apply the arsenic, as directed by Dr. Harris, but not leave it in over two hours; the decayed portion should then be removed, and the cavity washed out with the hydrated peroxyd of iron. This being an antidote to the arsenic, will arrest its further action.

It unfortunately so happens that the very class of teeth in which this article might be supposed to exert the most pernicious influence, are those in which it appears to be most demanded. And these are those teeth, apparently possessing a greater degree of vascularity than is usual, so that the sensi bility of the tooth bone is greater than is ordinarily met with. And I suppose that as is the vascularity of the dentine, so is the danger of reaching the pulp by the action of this remedy. The question, then, as I take it, assumes this character: Shall we let the tooth alone to be lost by the disease with which it is affected, or shall we attempt its preservation by the use of this remedy, and if the nerve should be destroyed, fill as if this had been done intentionally.

Although generally succeeding in the allaying of the extreme sensibility of these teeth, which are most frequently met with in youth, by those remedies first suggested, yet I must confess that they are not as prompt and certain in their action as is desirable, and the arsenic is too much so; taking the other extreme, various preparations I know have been recommended, such as sulph. ether and chloroform; the latter with benzoin, and various other remedies of the like nature. Yet to avoid pain in the removal of caries in the teeth of our young patients is of doubtful practicability. But I have digressed here farther than I intended, having reserved this subject for after consideration.

In the further consideration of this subject, we shall first take up the preparation of the cavities under consideration in the superior incisors and canine. And first, let us take a view as it were of the approximal surface of these teeth, so that we may have a proper idea of the form which the cavity will necessarily assume, particularly if the decay is at all extensive, and the separation of the teeth will demand the use of a file. In a majority of cases where the separation is effected by wedging, the cavities can be made round, or nearly so. But when the decay is extensive, commencing near the gum and extending to near the point of the tooth, we shall have a shape of cavity somewhat resembling the side view of the tooth itself. We have here wedged shape teeth, the palatal plane, however, far more beveling than the labial. The cavity, then, must often assume this shape; having its base next the gum, and the cone corresponding to the point of the tooth. The manner of separation will affect also the interior arrangement of the cavity. If, for instance, the teeth have been separated with the file, and the palatal portion most cut away to preserve the labial appearance of the teeth, we shall have a depth of cavity on the palatal, not at all equal to that on the labial line of the tooth.

This condition of things is generally that which is most desirable, so as to most effectually preserve the appearance of the tooth, and hide from view the filling.

The decay sometimes, however, approaches more the labial portion of the tooth. Then this condition of cavity may be somewhat reversed; and although the tooth may be well filled, yet the plug will be more exposed to view. The palatal border of these cavities do not always present a straight line, but generally the reverse, curving inward somewhat to the center of the decay.

The internal arrangement of these cavities I aim to get of the following order: The labial and palatal wall straight, merely carrying in as it were the breadth of the cavity at the orifice, but the base of the cavity next the gum, slightly enlarging as it extends in toward the center of the tooth. The point under the cutting edge of the tooth should also take somewhat this shape. In this way all the healthy bone which can *be*, should be left to give strength to the labial and palatal border or wall of the cavity, and this is especially necessary, as the palatal wall, if left thin, is apt to crumble away, and the labial may do the same, or reflect too much the gold filling.

It sometimes is necessary, however, to cut away from these points all the dentine, as this may have become discolored from disease. This enlargement of the cavity at the cutting edge and neck of the tooth, should not be of the character of a distinct neck, but merely such as to make a gradual widening of the cavity from these two points; so that the filling shall be held additionally secure. Scarce a tooth do we meet with which can be filled at all, but will bear this form of cavity; and in superficial or shallow cavities, it is that form most easily and securely filled. The remarks already made in relation to approximal cavities, are alike applicable to the incisors and canine superior and inferior. We come now to speak of those in the superior bicuspids. Here we have a different shape of tooth, and yet, as in almost all cases when the decay is small, the cavity is or may be easily made round. Here, however, wedges are not so much resorted to, and yet their use is often advantageous. I have of late years used them in all such cases as when the file would destroy the cavity. When these teeth have been filed and filled, the fillings are not doing well, or have dropped out, and the teeth are again pressing on each other. Such teeth I generally wedge apart—then shape my cavity and trim the border with a file to suit the condition of the teeth, so that two plugs may not press together, but allow the enamel portion on the labial face of the teeth to come in contact. Floss silk, or some such substance, should be daily forced between such teeth.

In separating these teeth where these conditions do not exist, I generally use the cutting instrument and the file. But let me here state one condition of things which we very often meet with in these teeth. We first have a crowded condition, then we have both bicuspids decayed on both approximal surfaces, and also decay on the anterior approximal surface of the first molar—the posterior bicuspids much decayed, and our patient sixteen. In my opinion, the best instrument to make space here is the forceps. One good space here is better than two, for this will be in a short time much diminished, and the annoyance to the patient in mastication, is far less than two filed spaces. This advice is made, of course, taking it for granted, that the molar and first bicuspid can be preserved.

Let us, however, prepare these teeth for filling without this operation. If the decay is advanced so far that the enamel has commenced to crumble in, I first take my cutting instrument, cut away that portion which is frail, and hides from view the cavity. This is done generally, cutting most toward the palatal portion of the tooth, preserving as much as possible the labial face of the tooth—the cavity is thus exposed—the depth of decay ascertained, and then with a knife-edged file the space is finished. This space, when completed, resembles somewhat the letter V, with its base toward the cutting point of the teeth; when admissible, a shoulder is left at the neck of the tooth. The space on the palatal line of these teeth is much wider than on the labial, when the decay is extensive, and the enamel frail, beveling from near the palatal prominence. This gives fine access to the cavity, and enables me to overcome a difficulty occasioned by an anatomical construction of the tooth, which is this: The fissure or suture running from approximal surface to approximal, as it approaches either surface, dips deep into the tooth; the center of this suture being elevated by the labial and palatal prominences uniting. This form of tooth, when the decay penetrates from the approximal surface of the tooth beneath this depression, and decomposes the bone to the enamel, weakens so much this exposed wall of the cavity, that it is constantly liable to be broken. The decay, following here the line of enamel, forms a shape of cavity any other than desirable for the retention of a plug. I prefer, therefore, at once to cut away the enamel to this depression, beveling in toward the palatal prominence, and up to the shoulder at the neck of the tooth. It also bevels toward the labial corner of the tooth, leaving the face of the tooth as perfect as the solidity of the enamel will allow. We have here a space giving us the most easy access to the cavity, and more easily kept clean with the tongue than any other.

The decay following the line marked out by the bone of the tooth, forms a circular cavity extending from under the labial to the palatal prominence, and the continuation of this circle interrupted by the depression on the cutting surface of the tooth. The depth of the cavity under the cutting surface of the tooth will necessarily be shallow, yet we will be enabled to cut under the enamel so as to get a good hold for the plug. The circle of the cavity first named can be so formed as to maintain the size of the cavity at its orifice, or, if desired, slightly enlarged, as described in my description of those in the central incisors. This shape of cavity at the neck of the tooth, I consider desirable in all approximal cavities; it holds in place the first blocks or fold of foil far better than any other, because the pressure made in compacting the gold is more on a direct line with the wall of cavity thus formed.

The space between the molars will, to a great extent, be the same as that already described, only the same care is not necessary to preserve the labial face of the tooth. If this decay is correspondingly large with that in the bicuspids, the space may also be wider, and as we advance back in the mouth, this will be needed, so that the cavity may be perfectly accessible.

Unless the decay dips beneath the margin of the gum at the neck of the tooth, the shoulder will be more distinct, and even when this is the case, the filling may be filed and trimmed to give the shoulder, almost as if the cavity did not pass under the gum. Without a good space and thorough work in the filling of these cavities, a difficulty so often complained of will be the almost certain result, and this is pain in picking the teeth. What occasions this pain? Is it, as is often said, an irritable periosteum, which has become exposed by the recession of the gum? I think this would not often give such acute pain as is complained of by the touch of the pick. Is it an exposure of the bone at the neck of the teeth, and which is firm and solid, and yet so sensitive? I think that this is not often the case.

This sensitive condition often comes on weeks or months after the tooth has been filled, and I think that it will more often be found to be the result of an imperfect removal of the diseased bone at the neck of the tooth, or the foil has not here been well compacted, so that moisture and air gets in and the bone beneath the filling becomos tender from disease. This I have frequently observed, and the refilling of the tooth relieved the difficulty. Very often have I found the space so small when this difficulty existed, that it was utterly impossible for any man to do justice to the operation. A narrow space between the molars will always be annoying and troublesome to the patient, while a very free opening is of but little if any inconvenience.

Some of the most difficult approximal cavities to fill are those on the anterior surface of the posterior molars, the anterior molar standing a little more prominent, and the posterior pressed against this by the dens sapientiæ. The cavity with an ordinary space is generally much hid from view, and that portion with its wall formed by the grinding surface of the tooth, completely so; so that the direction of force requisite to fill this portion of the cavity must be from the neck of the tooth; yet the pressure is most apt to be made just the reverse. I hope it will be borne in mind that I am pointing out difficulties not to the old and experienced operator. He has overcome them, but these are difficulties which every young operator will meet with, and the only way to overcome them is to take a firm stand, and so make your space as to have complete access to every portion of the cavity with your plugging instruments. The objections and foolish prejudices of the patient must not be heeded. Unless you can operate according to your own plan, you had better not operate at all.

In the larger class of approximal cavities, the drill will be found almost a useless instrument; the excavators alone being those we shall have to depend on to remove the decay and form the cavity; and these should be kept in most perfect order, having always on hand every form and variety which may be needed. For the formation of the cavity at the neck of the tooth, I like a simple curved excavator, flattened at the point with the curve of the instrument; the cutting edge of the instrument about as much beveled as I wish the wall of this part of the cavity to be; it may also be nearly as wide as the cavity is deep. The longest angle on the cutting edge of the instrument will be placed within the cavity, and by two or three cuts, circling with the neck of the tooth, mark out the depth you wish this wall of the cavity. These first cuts will line off the cavity so as not to expose the nerve by the future part of the operation; this instrument is then used in the same manner, until the shortest angle of the cutting edge trims away the entire decay from the border of the cavity.

These instruments will have to be in pairs, and made of different sizes, to correspond with the depth of decay. These instruments, with a variety bent at right angles with the bar of the instrument, some flattened so that their cutting edges shall be on a line with the bar, and others the reverse, will constitute those most used in the formation of these cavities.

In the smaller class of approximal cavities, the drill may be used, more, however, to give form to the cavity, than for the removal of the carious portion.

In approximal cavities, when space has been obtained by the removal of a tooth, the cutting instrument and file may not be necessary, unless, indeed, the border of the cavity is frail, or the enamel which covers the decay has not been broken in. If this should be the case, they will have to be used, but not to the same extent as where a tooth has not been removed. The border of the cavity should, however, always be firm and solid, not crumbling and breaking in under the pressure necessary for the consolidation of the gold.

A separate description of the preparation of approximal cavities in the inferior teeth, scarce requires any other description than that already given of the superior. The spaces to be made are the same between the molars, and but little difference between the bicuspids, except, indeed, not as a general rule, cutting so much from the palatal portion of these teeth.

When we come to the incisors, however, the same amount of filing is not admissible. The teeth are smaller, the cavities cannot penetrate very deep into the teeth, and much of the tooth removed from any cause would preclude the operation of filling without an exposure and destruction of the nerve. We find here, I think, the decay more frequently presenting to the labial face of the teeth. This enables us to fill frequently without any separation. When, however, much space is desirable, these teeth can be easily separated by wedging.

We come now to speak of the introduction of the foil in the filling of these cavities, and we take first the cavities in the inferior incisors. I stand at the right side and facing my patient, turning the face either to or from me, as the cavity may be in the approximal surface of the tooth nearest to, or farthest from me. If, for instance, it is the central left incisor, the cavity on the anterior approximal surface, the patient's face is slightly turned from me; a napkin folded over the tongue, and its folded corner brought around in front of the tooth, and is made to cover the gum and mucous membrane of the lip, which however, if possible, should be turned inward, and so held by the napkin, which is held in place by the forefinger of the left hand, with the thumb retaining the napkin in place over the tongue, and grasping the palatal surface of the tooth. The arm is thus thrown over or around the patient's head, and the three remaining fingers placed under the chin. The patient's head, chin, tongue and lip are now under my control.

The foil has been prepared by folding it from four to eight thicknesses, and cutting into a strip more than is sufficient to fill the cavity, but not as wide as the mouth of the cavity itself; a block is then folded on the end of this strip, as large as can well be placed endways in the cavity. The cavity being dried, this block or roll is with care adjusted and firmly compacted in that portion of the cavity nearest the gum. This block I introduce with my plugging forceps, and then take up a suitably curved and sharp pointed plugger, and with this, fold after fold of the strip is introduced. If the cavity is large, or large for one located in these teeth, I sometimes first introduce some two or three of these blocks, and then finish with a few folds of the gold. In the compacting and consolidation of the gold, the tooth is grasped firmly with the forefinger and thumb; a resisting force is thus applied to the tooth, and we are thus enabled to make the requisite pressure to consolidate the gold without injury to the tooth. After the last fold of gold has been well secured, I take my pointed plugger and pass it around the border of the cavity, compressing every point of the gold as I advance, and thus continue passing round, and each time nearing the center of the plug, until I have thus made pressure over the entire surface of the gold. I then take a flattened instrument, (and if the space is sufficiently large,) this is cut on its surface rough like a file, and with this make as much pressure as the size of the plug demands. The surface of the gold is then filed down and made level with the margin of the cavity. In this operation, I should always expect the solidity of the gold to be such by the time the last fold of gold was forced in, so that in passing my plugging instrument over the filling there will be more gold protruding than is possible to be forced into the cavity. This description will answer for the anterior approximal cavities in the inferior incisors and canine of the left side, and the posterior approximal of those of the right side. The insertion of the gold, indeed, in the other cavities of these teeth will be the same, but the manner of holding the tongue, lip, teeth, &c., will require a little explanation.

In the reverse cavities of these teeth, the patient's face is turned more toward me; the fold of the napkin is reversed, still keeping, however, the end next the lip. The forefinger of the left hand is placed on the napkin, over the tongue, pressing against the palatal face of the tooth, and sometimes the middle finger farther back on the tongue. The thumb is placed on the end of the napkin over the lip, and lays hold of the labial face of the tooth. This arrangement enables me to keep the tooth dry, and make counter pressure on the tooth when filling; and in adults, or with those who can maintain a fixed and firm position of the inferior maxillæ, the position and arrangement is a good one; but when support to the jaw appears to be more essential while introducing the gold, the forefinger may take the place of the thumb, the middle finger that of the forefinger, and the thumb thrown under the chin. After the foil has been introduced, the position of the left hand may be changed as desired, so as to most effectually aid in consolidating the plug. These teeth I generally fill from the outside. In irregularity of the teeth, an occasional exception occurs; the change of position, &c., must then be in accordance with the location of cavity, and for which I shall now give no special direction.

In the filling of the inferior bicuspids, I frequently use the napkin and left hand as above directed, especially when operating for young and unsteady patients, who cannot hold a firm jaw, and manage the speculum as I desire. I prefer, however, the use of the speculum when it is at all applicable, because I have the more free use of both hands in operating. Its application is much the same as in the filling of the central cavities of the inferior teeth, only the folds of the napkin should be more carefully held against the margin of the gum, and lying under the edge of the tongue opposite the tooth to be filled, and under the fold of the napkin a lock of cotton may be placed, to take up the saliva, and keep the cavity perfectly dry.

My position in the filling of these teeth, is to the right and in front of my patient, excepting in a few cases in the filling of the posterior approximal cavities, when I take my stand rather behind my patient, on a platform ten inches high, made as wide as my chair, the base of which my chair and foot-stool stand upon. The patient's head is thrown slightly forward, or held on a line with the body. Those cases where I should take my stand behind my patient, would be when the posterior approximal surface of either bicuspids, and sometimes the molars, had not been beveled sufficient to expose the cavity to view when standing in front. In cases of this kind, the cavities are not large, and I should prepare my gold as described for the inferior incisors, and with my plugging forceps, which are bent at near right angles, but when closed, flattened the reverse at the point from that used in the introduction of my blocks in the central cavities. After the block or roll has been pressed to its place, I either take up a bent plugger, adapted to such cavities, or use my forceps; taking hold of the strip far enough from its connection with the block introduced, so that when folded, and the point I have hold of is drawn into the bottom of the cavity, the end of the fold projects. This method is very accurate, and answers admirably until the cavity is nearly filled, when the forceps will have to be closed and used as a common plugger, or such an one selected. The last instrument used in this part of the operation should be small and sharp at the point. The operation thus far advanced, a flattened instrument which can pass between the teeth should now be used to force in as much of the projecting foil as is possible, and then, before the appliances for keeping the tooth dry are removed, pass a sharp pointed plugger over the surface of the gold, compressing and feeling of every point. In the use of the compressing instrument, the tooth being filled should be grasped with the thumb and forefinger of the left hand. If care has been taken in the introduction of the gold in cavities of this size, no point on the filling will admit the point of the sharpest plugger, so as to require any addition of foil. In small cavities where this is the case, I prefer refilling at once.

In the larger class of cavities now under consideration, a different procedure is somewhat necessary. The cavity is almost entirely filled with blocks, but as that portion of the cavity nearest the gum is deepest, owing to the beveling of the tooth, so the blocks used for this part should be the longest. We also find here the border of the cavity formed by the grinding surface of the tooth often depressed, so as necessarily to change the manner of introducing the gold. That portion of the cavity nearest the gum, however, should be first filled, and when the cavity has been filled to the depressed border, then that portion under the palatal prominence, and finish under the labial. This condition of things is, however, more observable in the superior bicuspids, yet often to be met with in the inferior bicuspids and molars. The folds of gold have been so carefully arranged in these blocks, that by proper care in their introduction, using considerable force in compacting as they are introduced, we shall have a solidity of gold hard to be procured in any other manner. I would here remark, that the shape of the cavity at the neck of the tooth, with its wall sloping inward, draws to the center of the cavities these blocks of gold. The pressure can then be made to the center of the tooth on an axis with its root, until we approach the last part of the operation in the introduction of the gold, when we come to wedge in, as it were, a few folds of foil under the wall of the cavity formed by the grinding surface of the tooth. Care should, however, be taken during the whole of the operation, to force into the bottom of the cavity the blocks of gold. In these large cavities, after I have made compression on the protruding ends of my blocks of gold, I examine carefully every point on the surface of the filling, and if a sharp pointed instrument can be introduced, it is done, and pressure made from this point to enlarge the opening; so that this becomes a cavity in shape to hold a plug, when a few folds of gold are introduced and compressed with the other.

When care has been taken in the introduction of the gold, and the border of the cavity has been sufficiently firm to resist the amount of pressure I usually make, I should not expect to make openings into the filling already introduced, for its more perfect consolidation. Yet, when the border of the cavity is necessarily frail, such may be the case; the instrument then should be introduced, not too near the weak wall of the cavity, but leave as much gold between it and the instrument as can be made solid without breaking it in. A few folds of gold will be a great protection, and prevent an injury to the cavity which might be irremediable.

Generally, the molar teeth are sufficiently firm to resist an amount of pressure necessary to consolidate the gold. When this is not the case, they should be held with the left hand of the operator. The pressure necessary to consolidate a plug, will depend on the size of the plug and the regular arrangement of the folds of gold in the cavity

The instruments I use for consolidating the gold in these large cavities, are bent at near right angles; some flattened with the bar of the instrument, and some the reverse. They are large and strong; the surface deeply cut like a coarse file, and will bear any amount of pressure which can safely be made on the tooth. After I have used these until I find the gold is solid and firm, I take a file, (one, perhaps, which has been partially worn out separating these teeth,) and file away that portion of the gold protruding beyond the margin of the cavity; but before this has been completely effected, I take a blunt pointed plugger, and make pressure entirely around the edge of the plug. Then use the file until it strikes on the enamel around the filling. We will still often have along the edge of the plug, next the grinding surface of the teeth, a fullness of the plug, occasioned by the curving in of this border of the cavity, which is the result of the depression on the grinding surface of the teeth already described; here, then, we take a small file, the blade of which is short and flattened, the cutting surface slightly rounded, the blade is bent with the handle to an angle of almost forty-five degrees; with this we bevel away the gold to correspond with this curved line of the border of the cavity. I would not be thus particular in this description, but these files are lately introduced, and are invaluable in cutting away the surplus gold of a plug.—*Dental Register.*

[To be continued.]

